# Are Blood Groups a Predictive Factor in Determining the Severity of Coronary Artery Disease in Patients Undergoing Coronary Heart Surgery?

**DOI:** 10.21470/1678-9741-2024-0280

**Published:** 2025-12-10

**Authors:** Mumtaz Murat Yardımcı, Cengiz Guven

**Affiliations:** 1 Department of Cardiovascular Surgery, Adıyaman University, Adıyaman, Turkey

**Keywords:** Th-Hr Blood-Group System, Taxus, Blood Group Antigens, Coronary Artery Bypass, Mortality, Percutaneous Coronary Intervention

## Abstract

**Objective:**

This study investigated whether blood groups are predictive factors for the severity and postoperative mortality in patients with coronary artery disease (CAD) undergoing bypass surgery with extracorporeal circulatory support

**Methods:**

A retrospective cohort study examined data from 4,002 patients who had coronary surgery for CAD between January 1^st^, 2014, and December 30^th^, 2020. The study recorded blood groups, demographic information, and and SYNergy between percutaneous coronary intervention with TAXus and cardiac surgery (SYNTAX) scores for patients who died within the first month post-operation.

**Results:**

Multiple regression analysis showed significant associations with the SYNTAX score (P < 0.001). Individuals with blood group O had a 2.970 times decrease in their SYNTAX score, while those with blood group A showed a 0.260 times increase, and those with blood group B had a 1.895 times decrease. Analyzing the effect of blood groups on mortality, the risk of death was significantly higher compared to blood group O; in group A the risk of death was 2.65 times higher than in group O (P = 0.005, odds ratio [OR]: 2.65, 95% confidence interval [CI]: 1.35 - 5.19). In group B the risk of death was 2.29 times higher than in group O (P = 0.048, OR: 2.29, 95% CI: 1.01 - 5.23). The Rh factor did not affect either mortality or CAD severity.

**Conclusion:**

In patients undergoing coronary surgery, the SYNTAX score was found to be significantly lower in blood groups O and B. However, regarding mortality, both blood groups A and B carried a higher risk of death when compared to group O.

## INTRODUCTION

**Table:** 

Abbreviations, Acronyms & Symbols				
ALT	= Alanine aminotransferase		LDL	= Low-density lipoprotein
AST	= Aspartate aminotransferase		MI	= Myocardial infarction
CABG	= Coronary artery bypass grafting		OR	= Odds ratio
CAD	= Coronary artery disease		SD	= Standard deviation
CI EF	= Confidence interval = Ejection fraction		SYNTAX	= SYNergy between percutaneous coronary intervention with TAXus and cardiac surgery
FBS	= Fasting blood sugar		TG	= Triglyceride
HDL	= High-density lipoprotein		VWF	= Von Willebrand factor

From the discovery of blood groups to the present day, it has been attempted to discover if the antigens that determine the groups might be among the determinants of hereditary diseases and, as a result, possible relationships between ABO groups and different diseases^[1,2]^.

Blood group antigens are expressed in many cells and tissues, especially platelets, as well as erythrocytes, which extended the clinical importance of blood groups beyond transfusion, and various studies suggested in the past that blood group antigens are an important factor in the development of different diseases. Data from scientific studies show that these antigens are associated with many organ tumors and coronary artery disease (CAD)^[3]^.

Many scientific studies reported in the past that the O blood group provides a protective effect against CAD and vascular diseases, while non-O blood groups show an increase in both the severity and prevalence of these diseases^[4-7]^.

There are also studies reporting positive associations between higher serum cholesterol levels and CAD in individuals other than group O^[3]^.

Despite the existence of all these reports, the relationship between blood groups and CAD leading to surgery has been significantly less investigated.

The present study reviewed the data of 4,002 patients retrospectively discussing the effects of blood groups on the severity of the disease and postoperative mortality in operated CAD patients based on literature data.

## METHODS

The present study has a retrospective and cohort design. Our hospital’s Ethics Committee approved the study (with decision no:03 on 23.01.2023). A total of 4,002 patients who underwent coronary artery bypass grafting (CABG) surgery with cardiopulmonary bypass between January 1^st^, 2014 and December 30^th^, 2020 were included in the study. The data were collected retrospectively from the hospital’s patient record system. Clinical and laboratory data of the patients were extracted and recorded, and SYNergy between percutaneous coronary intervention with TAXus and cardiac surgery (SYNTAX) scores were calculated. Blood groups were determined. Patients who died before discharge were identified. SYNTAX scores were calculated separately and compared to determine the prevalence and severity of CAD. The data of the deceased patients were examined, and the possible effect of blood groups on mortality was investigated.

Patients who underwent elective surgery and did not have an active infection, hyper/hypothyroidism, or malignancy that would prevent surgery were included in the study. A total of 461 patients were excluded because they met the exclusion criteria. These included 57 patients who underwent CABG + valve surgery, 395 patients who underwent one or more valve surgeries, two patients with diagnosed severe liver disease (cirrhosis or malignancy), and seven patients with end-stage renal disease or on dialysis.

The IBM SPSS Statistics for Windows, version 20.0 (IBM Corp., Armonk, N.Y., USA) was used in the analyses. The Shapiro-Wilk normality test of variables and Mann-Whitney U test were used in comparisons, and the Chi-square test was used in categorical variables. Factors affecting mortality and SYNTAX score were examined with univariate and multivariate regression analysis. The Bonferroni correction was used to control false positive results when testing the effect of more than one independent variable. The significance level was taken as *P* < 0.05.

## RESULTS

A total of 4,002 patients were examined through scanning the files (1906 [47.6%] were male and 2096 [52.4%] were female). The mean age was found to be 60.5 ± 10.4 years in males and 59.8 ± 10.4 years in females. ABO distribution was: 1,306 (32.6%) of the patients were in group O, 1,772 (44.3%) in group A, 628 (15.7%) in group B, and 296 (7.4%) in group AB. Preoperative demographic characteristics are summarized in Table 1.

**Table 1:** Demographic and laboratory data of patients, mean ± standard deviation.

	O	A	B	AB	*P*-value^a^
n (%)	1306 (32.6)	1772 (44.3)	628 (15.7)	296 (7.4)	
Age, years	60.2 ± 10.8	60.1 ± 10.1	60.3 ± 10.3	60.3 ± 10.7	0.764
Sex (n)					
Male	653	817	289	153	0.567
Female	655	953	339	143	
EF	54 ± 9	54.2 ± 9	53.4 ± 9.5	53.9 ± 9	0.404
SYNTAX	34.5 ± 18.3	37.5 ± 6	35.5 ± 9	37 ± 5.4	0.001
FBS	138.7 ± 75.8	136.6 ± 70.3	134.7 ± 69.3	137.2 ± 67.9	0.413
HbA1C	6.1 ± 2.2	6.1 ± 3.8	5.9 ± 1.9	6.1 ± 2.1	0.526
Cholesterol	163.4 ± 38.7	177.4 ± 46.3	168.1 ± 34.5	163.9 ± 40.9	0.068
TG	159.4 ± 94	107.3 ± 111.7	162.5 ± 94.3	157.3 ± 102.2	0.851
HDL	45.3 ± 11.7	75.5 ± 12.2	45.3 ± 11.8	46.4 ± 13.1	0.150
LDL	86.3 ± 34.8	71.6 ± 38.6	90.3 ± 29.8	86.0 ± 35.9	0.123
Urea	37.8 ± 17	37 ± 16	39 ± 18.1	37 ± 16.6	0.818
Creatinine	0.9 ± 0.3	0.9 ± 03	0.9 ± 03	0.9 ± 0.3	0.726
ALT	22.8 ± 15.8	22.5 ± 16.8	21.6 ± 13.5	23.9 ± 18.7	0.866
AST	24 ± 18.8	24.7 ± 20.4	25.7 ± 23	26.1 ± 23.2	0.039

a Chi-square test

ALT=alanine aminotransferase; AST=aspartate aminotransferase; EF=ejection fraction; FBS=fasting blood sugar; HDL=high-density lipoprotein; LDL=low-density lipoprotein; SYNTAX=SYNergy between percutaneous coronary intervention with TAXus and cardiac surgery; TG=triglyceride

Based on the file scan, it was understood that 68 (1.7%) of the 4,002 patients died. In the Mann-Whitney U test used to compare variables between deceased and living patients, high ejection fraction (EF), low SYNTAX score, low HbA1C level, and low triglyceride (TG) were associated with reduced mortality (*P* < 0.001, *P* < 0.001, *P* < 0.001, and *P* = 0.045, respectively) (Table 2).

**Table 2:** Factors affecting mortality.

Variables	Alive	Died	*P*-value^a^
	(n = 3933)	(n = 68)	
Age, years	61 (14)	61 (11)	0.511
EF	56 (14)	45 (14)	< 0.001^*^
CABG	3 (1)	3 (1)	0.120
SYNTAX	34 (12)	52 (8)	< 0.001^*^
HbA1C	5.2 (1.3)	6.7 (5.9)	< 0.001^*^
Cholesterol	181.8 (56.6)	185.8 (73.6)	0.621
TG	142 (109)	158 (116)	0.045^*^
HDL	44 (15)	44.5 (15.5)	0.437
LDL	104 (50)	106 (49.8)	0.762
Urea	33 (18)	31 (18)	0.479
Creatinine	0.83 (0.3)	0.79 (0.2)	0.675
AST	19 (13)	19.5 (15)	0.844
ALT	20 (13)	20 (15.5)	0.709

* *P* < 0.05 significance level; median was used to define the variable;

a Mann-Whitney U test

AST=aspartate aminotransferase; ALT=alanine aminotransferase; CABG=coronary artery bypass grafting; EF=ejection fraction; HDL=high-density lipoprotein; LDL=low-density lipoprotein; SYNTAX=SYNergy between percutaneous coronary intervention with TAXus and cardiac surgery; TG=triglyceride

Logistic regression analysis was used between the factors affecting mortality in Table 2. Each unit decrease in EF increases the risk of mortality by 7% (OR: 0.93) (*P* < 0.001). Each unit increase in SYNTAX score increases the risk of mortality by 12% (OR: 1.12) (*P* < 0.001), and each unit increase in HbA1C increases the risk of mortality by 4% (OR: 1.04) (*P* = 0.023). TG levels did not have a significant effect on mortality (*P* = 0.313) (Table 3).

**Table 3:** Determination of risk factors affecting mortality.

Factor of risk	OR (95% CI)	*P*-value
EF	0.93 (0.90 - 0.95)	< 0.001
SYNTAX	1.12 (1.09 - 1.14)	< 0.001
HbA1C	1.04 (1.01 - 1.08)	0.023
TG	1.001 (0.999 - 1.003)	0.313
Blood group (reference category: O)		
A	2.65 (1.35 - 5.19)	0.005
B	2.29 (1.01 - 5.23)	0.048
AB	2.44 (0.89 - 6.64)	0.082

Logistic regression model was found to be significant (*P* < 0.001) CI=confidence interval; EF=ejection fraction; OR=odds ratio; SYNTAX=SYNergy between percutaneous coronary intervention with TAXus and cardiac surgery; TG=triglyceride

In Table 3, A, B, and AB blood groups are associated with mortality risk compared to the reference category blood group O. It is seen that the A, B, and AB blood groups increase the mortality risk by 2.65, 2.29, and 2.44 times, respectively, compared to the blood group O. This relationship is statistically significant for A and B blood groups (*P* < 0.05). However, significance was not reached for the AB blood group (*P* = 0.082).

In the variance analysis used between the variables in Table 4, a significant difference was detected between the blood groups in terms of SYNTAX value (*P* < 0.001). In the pairwise comparison used to determine the difference, it was found that the O blood group had a significantly lower SYNTAX score than the A, B, and AB blood groups (*P* < 0.001). A difference was also observed between the A and B blood groups in terms of SYNTAX value (*P* < 0.001). The A blood group had a higher SYNTAX score. Finally, the B and AB blood groups showed a difference in SYNTAX value (*P* < 0.001), and the B blood group had a lower SYNTAX score. The median SYNTAX value was the lowest in the O blood group. There was no significant difference between the Rh groups in terms of SYNTAX value (*P* = 0.427).

**Table 4:** Comparison of SYNTAX values between blood groups.

Variables	SYNTAX	*P*-value	Dual comparison
Blood group		< 0.001^*^	*P*^O-A^: < 0.001
O (n = 1306)	27 (37)		*P*^O-B^: < 0.001
A (n = 1772)	35 (9)		*P*^O-AB^: < 0.001
B (n = 628)	35 (11)		*P*^A-B^: < 0.001
AB (n = 296)	36 (5)		*P*^A-AB^: 0.758
			*P*^B-AB^: < 0.001
Rh		0.427	-
Rh (-) (n = 368)	35 (12)		
Rh (+) (n = 3634)	35 (12)		
SYNTAX=SYNergy between percutaneous coronary intervention with TAXus and cardiac surgery			

The effects of blood groups on SYNTAX value were examined by the multiple linear regression analysis. Based on the analysis results, the O and B blood groups had a statistically significant and negative (low SYNTAX score) effect on SYNTAX score (*P* < 0.001 and *P* < 0.001, respectively), while the blood group A had a positive and significant (high SYNTAX score) effect (*P* = 0.024). Blood group AB had no significant effect on SYNTAX score (*P* = 0.508) (Table 5).

**Table 5:** Examining the SYNTAX effects of blood groups.

Dependent variable (SYNTAX)	β	SD	*t*	*P*-value	95% CI	
					Lower	Upper
Constant	37.465	0.281	133.171	< 0.001	36.913	38.017
O	-2.970	0.432	-6.878	< 0.001	-3.817	-2.124
A	0.260	0.115	5.11	0.024	0.035	0.486
B	-1.895	0.550	-3.446	< 0.001	-2.973	-0.817
AB	-0.492	0.744	-0.662	0.508	-1.950	0.966

Model R^2^ = 0.111; Model significance = 0.001

CI=Confidence interval; SD=standard deviation; SYNTAX=SYNergy between percutaneous coronary intervention with TAXus and cardiac surgery

When the Rh factor was evaluated together with blood groups (*e.g.*, ORh+, ORh-), its effect on the SYNTAX score was not found to be statistically significant (*P* = 0.427). The overall significance of the model was determined as *P* < 0.001, and the R^2^ value was 0.661.

## DISCUSSION

A scoring system called the SYNTAX score was developed in 2005 to determine the treatment options for complex CAD. Increasing SYNTAX score is directly proportional to increasing mortality and CAD severity^[8]^.

In the present study, assuming that increasing SYNTAX score is determined by increasing CAD severity, there was decreased CAD severity in individuals with blood groups O and B compared to blood group A (more in blood group O). There was also a higher mortality rate in blood group B compared to blood group O. In our study of individuals undergoing CABG, blood group O was associated with lower mortality, while blood group A had higher mortality rates. Also, in the results of the present study, low EF, presence of diabetes mellitus and high TG were found to be associated with mortality and CAD severity.

The carbohydrate structures that make up the ABO blood group were identified in various cell types, including platelets and endothelial cells. ABO antigens are the terminal sugar structures of glycan chains. The A and B alleles at the ABO locus on chromosome 9 express either A- or B-glycosyltransferase enzymes, which catalyze the addition of specific sugar residues to convert the core structures to form either the A antigen or the B antigen. As a result, the A and B structures differ only in a single terminal sugar moiety (N-acetylgalactosamine and D-galactose). Individuals in group O have no A-or B-transferase activity and thereby continue to express the basic glycan structure at the ends of their oligosaccharide chains^[9]^.

A literature review showed an association between blood groups and several autoimmune diseases, such as type 1 diabetes, psoriasis, multiple sclerosis, and Crohn’s disease, as well as many pathological conditions, such as congenital heart disease and CAD^[10-15]^.

A previous study reported the links between thromboembolic events and blood groups. Ischemic cardiovascular events, myocardial infarction (MI), atherosclerotic vascular disease, and venous thromboembolism were all found to be linked to each other, and all cases were found to be higher in non-O blood group individuals^[16]^.

In the present study, the presence of blood group O was shown to be protective against CAD severity and mortality. In our study, unlike the literature data, the blood group B was also associated with lower CAD severity compared to the blood group A. Acar et al.^[17]^ reported a significant relationship between idiopathic atrioventricular block and blood groups. Gostman et al.^[18]^ found that blood groups have prognostic value in patients with heart failure. In the present study, no relationships were detected between the Rh factor and CAD severity and mortality. Rh factor did not cause any significant change in both mortality and SYNTAX score (CAD severity). Huang et al.^[19]^ reported that individuals with blood type O had greater plaque stabilization than individuals without blood group O, and that individuals without blood group O were more prone to CAD.

In a different study that was conducted with the Chinese population, it was speculated that ABO genetic variations might contribute to large artery atherosclerosis but did not affect small vessel diseases and ischemic stroke^[20]^. In another study, it was reported that higher arachidonic acid levels were required for platelet aggregation in individuals with blood group O treated with acetylsalicylic acid and oral P2Y12 receptor inhibitors (such as clopidogrel, prasugrel, ticagrelor, ticlopidine), and a higher platelet reactivity index was observed in blood group A^[21]^.

In a study similar to ours, no association was detected in individuals who underwent CABG between blood groups and the prevalence of CAD, whereas group B was shown to have higher rates of MI, lower extremity ischemia, stroke, and need for urgent revascularization. In our study, blood group B was associated with lower CAD severity than blood group A^[22]^. Von Willebrand factor (VWF) ensures platelet adhesion in endothelial damage and acts as a carrier protein for factor VIII. In this way, it increases the half-life of factor VIII by six-fold. Increased levels of VWF are detected in venous and arterial thrombosis. The main determinant of VWF levels in circulation is the ABO blood group. It has been shown that levels increase by 25 - 30% in individuals without O blood group^[23]^.

Previous studies conducted on the effect of the blood group AB on CAD have yielded conflicting results. Although some studies suggest that the blood group AB increases the risk of CAD, some publications found no significant differences between individuals with the AB blood group and those without it. Furthermore, many studies have focused on blood group O and found that non-O blood groups increase the severity of CAD and mortality compared to the blood group O^[24-26]^. In the present study, the AB blood group could not be statistically calculated because of insufficient data in the model we established. Larger and multicenter studies are needed to understand the effects of the AB blood group.

ABO blood groups are defined as non-modifiable risk factors in this study and many other publications. Knowing the effects of ABO blood groups on mortality in CAD might reduce mortality in patients undergoing coronary surgery by changing modifiable risk factors. For example, significant reductions in mortality can be achieved in patients at risk with appropriate exercise programs, smoking cessation, and appropriate diets.

The results of the present study, which was conducted with the help of a literature review, show that individuals without blood group O are associated with many pathological conditions, especially acute MI and atherosclerotic cardiovascular disease. Although the exact mechanism is not known, it seems to be the ABO modification of complex molecules on the platelet surface that affects platelet functions. In this regard, larger-scale and multicenter studies are needed to better understand all genomic modifications.

### Limitations

There were some limitations to the present study. First of all, the study had a retrospective design. The relatively low number of cases might have affected the results. In the multivariate logistic regression analysis used to examine the effects of blood groups on SYNTAX score, the low R2value of the model emphasized that other factors (genetics, environmental factors, education, etc.) should also be examined. Another limitation was that the study was single-centered, and our study group consisted mostly of Turkish race. Since it is known that ABO blood groups are distributed according to ethnicity, this may also have affected the results. More comprehensive future studies, that have different ethnic origins and are multi-centered, will enable us to reach more precise and accurate conclusions. Another limitation was that mortality was examined up to 30 days (one month) in patients who underwent CABG. Looking at later times could have provided us with different data.

## CONCLUSION

In conclusion, based on the results of the present study, blood groups O and B (more in blood group O) provided a protective effect against the severity of CAD, but increased CAD severity was found in individuals with blood group A. Non-O blood groups were associated with increased mortality. The relationship between the Rh factor and CAD mortality and severity could not be demonstrated.

## Data Availability

The author declares that he does not have a special link for data repository but is ready to share it.

## Artificial Intelligence Usage

The authors declare that no artificial intelligence tool was used in the preparation of this article.
